# Assessment of a quantitative metric for 4D CT artifact evaluation by observer consensus

**DOI:** 10.1120/jacmp.v15i3.4718

**Published:** 2014-05-08

**Authors:** Sarah J. Castillo, Richard Castillo, Peter Balter, Tinsu Pan, Geoffrey Ibbott, Brian Hobbs, Ying Yuan, Thomas Guerrero

**Affiliations:** ^1^ The University of Texas Health Science Center Houston TX; ^2^ Divisions of Radiation Oncology The University of Texas MD Anderson Cancer Center Houston TX; ^3^ Diagnostic Imaging The University of Texas MD Anderson Cancer Center Houston TX; ^4^ Quantitative Sciences The University of Texas MD Anderson Cancer Center Houston TX; ^5^ Department of Computational and Applied Mathematics Rice University Houston TX USA

**Keywords:** 4D CT, artifacts, correlation, visual assessment

## Abstract

The benefits of four‐dimensional computed tomography (4D CT) are limited by the presence of artifacts that remain difficult to quantify. A correlation‐based metric previously proposed for ciné 4D CT artifact identification was further validated as an independent artifact evaluator by using a novel qualitative assessment featuring a group of observers reaching a consensus decision on artifact location and magnitude. The consensus group evaluated ten ciné 4D CT scans for artifacts over each breathing phase of coronal lung views assuming one artifact per couch location. Each artifact was assigned a magnitude score of 1‐5, 1 indicating lowest severity and 5 indicating highest severity. Consensus group results served as the ground truth for assessment of the correlation metric. The ten patients were split into two cohorts; cohort 1 generated an artifact identification threshold derived from receiver operating characteristic analysis using the Youden Index, while cohort 2 generated sensitivity and specificity values from application of the artifact threshold. The Pearson correlation coefficient was calculated between the correlation metric values and the consensus group scores for both cohorts. The average sensitivity and specificity values found with application of the artifact threshold were 0.703 and 0.476, respectively. The correlation coefficients of artifact magnitudes for cohort 1 and 2 were 0.80 and 0.61, respectively, (*p* < 0.001 for both); these correlation coefficients included a few scans with only two of the five possible magnitude scores. Artifact incidence was associated with breathing phase (*p* < 0.002), with presentation less likely near maximum exhale. Overall, the correlation metric allowed accurate and automated artifact identification. The consensus group evaluation resulted in efficient qualitative scoring, reduced interobserver variation, and provided consistent identification of artifact location and magnitudes.

PACS numbers: 87.57.qp, 87.55.Gh, 87.57.C‐

## I. INTRODUCTION

Four‐dimensional computed tomography (4D CT) is a common method of radiation therapy simulation used to account for respiratory motion in treatment planning.^1^, ^2^, ^3^, ^4^, ^5^ Four‐dimensional CT has allowed reduction of motion artifacts and systematic uncertainty in treatment planning, enabling smaller target margins.^6^, ^7^, ^8^, ^9^ Two implementations of 4D CT are commonly used: ciné 4D CT^10^, ^11^ and helical 4D CT.^8^, ^11^, ^12^

Ciné 4D CT is an axial mode of CT that acquires discrete segments of multislice images across the scan extent, while helical 4D CT uses a low pitch across the scan extent. Each implementation ideally acquires each imaged voxel for at least one breathing cycle. Breathing information is derived from an independent external surrogate for internal motion that provides a one‐dimensional respiratory trace during image acquisition. This allows images to be sorted into multiple, typically ten, 3D CT scans, each representing a breathing phase. This study focused on ciné 4D CT, which sorts phases on the basis of a nearest‐neighbor technique, typically phase sorting, that finds the image segment closest in time to each externally derived breathing phase at each couch position.^2^, ^13^

**Figure 1:**
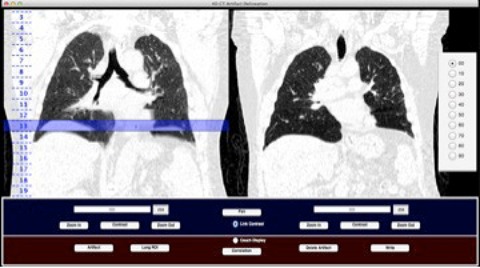
Artifact evaluation software showing: T0% of a 4D CT scan (left) with a highlighted identified artifact at couch position 13 indicating a saved artifact location; and corresponding deep‐inspiration breath‐hold scan (right) used as an anatomic reference for artifact identification in the 4D CT (left).

The ciné acquisition and phase sorting of discrete image segments often result in coronal and sagittal views that exhibit artifacts (see Fig. 1) in the form of discontinuous bands of image segments between couch positions. Artifacts, or artificial anatomic spatial distributions, cause uncertainty in the true anatomic spatial distribution and errors in anatomic delineation, treatment targeting, and lung function images derived from CT.^14^, ^15^, ^16^, ^17^, ^18^, ^19^ Therefore, many studies have explored methods to evaluate and reduce the frequency and magnitude of 4D CT artifacts.

Cui et al.^(20)^ devised a correlation metric (CM) for assessment of ciné artifacts that attempts to account for normal anatomic variations. This metric correlates the image slices across two neighboring couch positions and subtracts the average of correlations between adjacent image slices within the neighboring couch positions. This is a straightforward, efficient metric that can potentially distinguish normal anatomic variation from artifact and is very promising. However, the metric's validation was limited to relative metric values between ten 4D CT scans with two observers. Validation of this metric can be improved by a receiver operating characteristic (ROC) analysis^21^, ^22^ to assess the metric's accuracy in artifact identification.

Han et al.^(23)^ also explored use of a correlation metric for artifact indication on helical scans using a reference ‘bridge stack’ of images overlapping in anatomy at different breathing phases. This method has advantages of automation and efficiency, but requires use of an anatomic reference that is derived from the 4D CT scan and, therefore, subject to the artifacts being attempted to quantify. The current standard of artifact evaluation is manual visual assessment, which is associated with high interobserver variability, lack of quantitative power, and lengthy analysis times. A consensus group of expert observers may reduce analysis times and interobserver variation, yielding a higher quality standard evaluation dataset.

In this study, a consensus group evaluated ciné artifacts in ten 4D CT scans to form a standard dataset. This dataset was then used to assess the performance of the correlation metric in artifact identification^(20)^ through ROC analysis.

## II. MATERIALS AND METHODS

### A. The proposed metric

The metric proposed by Cui et al.^(20)^ is based on a normalized cross‐correlation (NCC) coefficient between two axial images (Eq. (1)). This coefficient is commonly used in template matching to determine the position of a given pattern in an image.^(24)^ The position of a template *t* within an image f(x,y) is given by (u,v), where $\bar{t}$ is the mean of the template pixel intensities and ${\bar{f}}_{u,v}$ is the mean of f(x,y) pixel intensities in the region under the template. The maximum NCC value gives the position match.

(1) $$NCC=\frac{\sum_{x,y}\left([f(x,y)-{\bar{f}}_{u,v}][t(x-u,y-v)-\bar{t}]\right)}{{\left(\sum_{x,y}{\left[f(x,y)-{\bar{f}}_{u,v}\right]}^{2}\sum_{x,y}{\left[t(x-u,y-v)-\bar{t}\right]}^{2}\right)}^{0.5}}$$

The Pearson correlation coefficient C is typically used in correlation studies to assess a linear relationship between two variables or images. The two‐dimensional (2D) Pearson correlation coefficient was used in this study (Eq. (2)) for efficiency of correlating an image A with a second image B, where $\bar{A}$ is the mean of image *A* pixel intensities, $\bar{B}$ is the mean of image *B* pixel intensities, and (m,n) are indices of pixel rows and columns, respectively. The Pearson correlation coefficient C is equal to the maximum NCC coefficient when the two images are properly aligned. We chose to use the Pearson correlation coefficient rather than the NCC coefficient because the Pearson correlation coefficient requires less computation time and is more intuitively understood.

(2) $$C=\frac{\sum_{m}\sum_{n}\left(A_{mn}-\bar{A})(B_{mn}-\bar{B}\right)}{\sqrt{(\sum_{m}\sum_{n}{\left(A_{mn}-\bar{A}\right)}^{2})(\sum_{m}\sum_{n}{\left(B_{mn}-\bar{B}\right)}^{2})}}$$

All calculations were performed using MATLAB (The MathWorks, Inc., Natick, MA) with the *corr2* function (Eq. (2)) for the correlations. The correlation metric devised by Cui et al.^(20)^ (Eq. (3)) was calculated between each couch position N per breathing phase per 4D CT scan. A couch position is a reference to a beam‐width size superior‐inferior location across the scan extent; each couch position contains a sorted image segment with 8×2.5 mm thick axial images. The Pearson correlation coefficient C was calculated between image 7 and image 8 of couch position $N(C_{7,8}^{N})$, then between image 1 and image 2 of the inferior couch position $N+1\;(C_{1,2}^{N+1})$; the resulting two coefficients were averaged to account for normal anatomic variation. The coefficient between image 8 of couch position N and image 1 of couch position $N+1\;(C_{8,1}^{N,N+1})$ was subtracted from this average, yielding final metric CM. A lower CM value indicates a better image match, and a lower artifact severity. Each 4D CT scan had a corresponding matrix of $\left((N_{T-1})\times 10\right)$ CM values, where $N_{T}$ is the total number of couch positions per each of 10 phases.

(3) $$CM=0.5(C_{7,8}^{N}+C_{1,2}^{N+1})-C_{8,1}^{N,N+1}$$

One metric per each couch position 2 through $N_{T}‐1$ was needed for comparison with observer results, so each couch position's bordering CM values were averaged to mimic how an observer would assess a couch position for artifact presence. A final normalized correlation metric (NCM) was calculated by dividing each CM value by the average of CM values within that phase. This normalization allowed retention of the relative values for comparison between 4D CT scans and between phases, while yielding a common reference point for 4D CT scans.

### B. The sample evaluated

We identified ten patients scheduled to undergo thoracic radiation therapy at our institution. Our study sample consisted of the clinical ciné 4D CT scans used for each identified patient's radiation treatment plan. All 4D CT images were acquired using a GE Discovery ST PET/CT scanner (GE Medical Systems, Waukesha, WI), with the 8‐slice LightSpeed CT component, and retrospectively reviewed. All image segments were 8×2.5 mm slices with an × and y voxel size of 0.97 mm. Phases were binned evenly in time between user‐defined maximum inhalation peaks for all cases. Maximum inhale is represented by T0%, with each subsequent breathing phase per cycle defined in 10% increments of the breathing period for that cycle (i.e., T0%, T10%, T20%, etc.). All 4D CT scans were phase‐sorted with this binning technique using the GE Advantage Workstation software (GE Medical Systems).

A sample size calculation estimated the minimal number of 4D CT scans necessary for metric evaluation. Two cohorts were needed, one to determine a metric threshold for artifact identification and a second to evaluate the determined artifact threshold. We calculated an exact test for single proportion (Eq. (4)) to determine the sample size with the software nQuery Advisor (Statistical Solutions, Boston, MA). Given × successes out of n trials, x=0,1,…,n, the p‐value for an exact, two‐sided binomial test under null success rate $\varphi_{0}$ is computed by summing the probability of observing values of the sample space that are as or more extreme than x.

(4) $$\sum_{u=0}^{x}\frac{n!}{u!(n-u)!}\pi_{0}^{u}{\left(1-\pi_{0}\right)}^{n-u}+\sum_{u=n-x-1}^{n}\frac{n!}{u!(n-u)!}\pi_{0}^{u}{\left(1-\pi_{0}\right)}^{n-u}$$

A test significance alpha level of 0.1 was used with type 1 error and a power of 0.81. A twosided null hypothesis of 0.5 with an alternative of 0.75 was chosen, indicating the metric could not definitively identify an artifact or that it could identify at least three artifacts of every four artifacts present. This yielded 26 artifacts needed per cohort. If at least one artifact exists per breathing phase per 4D CT scan, then a single 4D CT scan will contain at least ten artifacts. Therefore, five 4D CT scans per cohort were evaluated, approximately double the number of artifacts needed. Each 4D CT scan was briefly visually assessed to ensure this minimum artifact requirement was met.

### C. observer artifact assessment

Thorough visual assessment is time‐consuming and associated with high interobserver variance,^(25)^ but remains the standard artifact evaluation method. To reduce both the time required and interobserver variance, we organized a committee of observers to view images simultaneously and reach a consensus on artifact location and magnitude. We termed this committee the consensus group. The consensus group consisted of a physician specializing in thoracic oncology, a physics assistant working with ciné 4D CT, two physicists in the thoracic service, and a dosimetrist who works with thoracic treatment plans. An independent member, a graduate student studying 4D CT, coordinated each assessment and distributed materials necessary for evaluation, but did not participate in the actual assessment.

Prior to the first consensus group meeting, instructions for artifact evaluation were given to all consensus group members. The same instructions were provided at each consensus group meeting. The instructions included definitions and examples of ciné 4D CT artifacts with our magnitude scoring system, as well as an example of a cardiac artifact that results from the heart beating asynchronously from the breathing motion of the lung that may potentially be mistaken for a 4D CT artifact. These instructions served as a baseline reference to calibrate each observer's visual scale.

**Figure 2:**
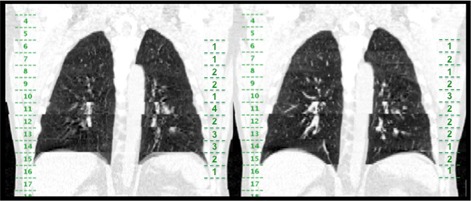
Example of coronal slices taken from T10% (left) and T90% (right) of patient $C2_{\_}1$ (NCM values shown in Fig. 3.), with couch positions indicated on the left side of each coronal view and consensus group identified scores per couch position shown on the right side of each coronal view. The artifact in T10% at couch position 11 was scored as a more severe artifact based on the higher interference of the artifact with anatomy.

If a couch position within a breathing phase was identified as containing an artifact, it was assumed that the identified artifact existed at all image slices at that couch position within that phase. All couch positions covering lung anatomy were assessed; couch positions below the displayed inferior lung were considered in assessment if there appeared to be an artifact at that location interfering with display of what should have been the most inferior lung anatomy. Each identified artifact was assigned a magnitude score between 1 and 5, with 1 indicating an artifact with a minor degree of interference with true anatomy and 5 indicating a large degree of interference with true anatomy; Fig. 2 gives an example of artifact scores assigned. Coronal views were assessed in all 10 phases of each patient. A helical deep‐inspiration, breath‐hold scan acquired within the same examination as the 4D CT scan was displayed next to the 4D CT scan during assessment to serve as an anatomic reference as it is free of the ciné 4D CT artifacts being evaluated. Artifacts were assessed using custom MATLAB software that allowed simultaneous coronal display of a component breathing phase of the 4D CT and the coronal display of the breath‐hold. This software also allowed the ability to scroll through the images, change the window and level, zoom in or out, display the couch position locations, change the breathing phase, and save identified artifact's corresponding couch position and score (Fig. 1).

Before the 4D CT evaluation, a lung window of level ‐450 HU and window 1100 HU was set for both the breath‐hold scan and the 4D CT scan. Each was zoomed so that the entire lung with an extra couple of surrounding couch positions could be viewed. The couch positions of the 4D CT scan were displayed after the zoom so that observers could identify correct couch positions. Two sets of numbered cards were distributed to each observer, and the observers used these cards to identify couch positions and scores for artifacts independent of the other observers' choices.

The breath‐hold scan was scrolled through before each 4D CT evaluation for anatomic ref, and then the entire lung in the selected 4D CT breathing phase was scrolled through, allowing time for observers to choose their numbered cards for artifact location and score. Then for each couch position within the lung, the consensus group was asked if the stated couch position contained an artifact. Each observer shared the numbered cards he or she had chosen. Results were saved if all consensus members agreed on an artifact location and score; if different answers were chosen, the observers made a case for their reasoning and the images were reviewed until the group achieved a consensus. Only rarely was no consensus reached; in these cases the majority ruled.

#### C.1 Statistical analysis of observer artifact assessment

A logistic mixed effects model was used to evaluate the relationship between artifact incidence and breathing phase from consensus results from all ten patient cases using the lme4 package in R.2.14.0 (http://www.r‐project.org/). The utilized mixed logistic model facilitates joint estimation of the log‐odds of artifact incidence across all phases, while accounting for the inherent correlation among observations derived from the same patient scan, to estimate and characterize the extent of variability associated with breathing phase. The likelihood ratio test for association is reported; this test is used to weigh the observed evidence of homogeneity (i.e., the identical log‐odds for all phases). In addition, phase effects were evaluated for significance using twosided Wald tests. Wald tests are used to weigh the observed evidence of phase‐specific log‐odds adjustments from component phase T0% in the presence of the estimated within and between subject sources of variability. Post hoc application of the sequentially rejective Bonferroni method was used to adjust p‐values for multiple comparisons of the adjustments of log‐odds of artifact incidence from component phase T0% across phases T10% through T90%.^(26)^ The multiple phase comparisons inflate the nominal false positive rate, and thus p‐values need to be adjusted to maintain the type I error rate at 5%.

### D. Artifact threshold derivation and evaluation

An ROC method was used to determine an artifact threshold with cohort 1; this artifact threshold was then applied to cohort 2 for evaluation of resulting sensitivity and specificity. The artifact threshold is a NCM value. A 4D CT contains NCM values for each component breathing phase at each couch position except the first and last as given in the matrix $\left((N_{T-2})\times 10\right)$. The NCM values above or equal to the applied artifact threshold value will indicate an artifact at an anatomic location (N) and breathing phase. MATLAB software was developed to format observer results and produce ROC curves. In order to generate ROC curves, a binary decision threshold is moved across the data, above which an artifact is identified, below which an artifact is not identified; each decision threshold yields a sensitivity and false‐positive fraction point on the curve.^(27)^ The decision threshold was incremented by 1% between the minimum NCM value and the maximum NCM value contained within each cohort 1 patient $\left((N_{T-2})\times 10\right)$ matrix. As the decision threshold was incremented, NCM values below the decision threshold were set to zero, indicating no artifact presence. The consensus results served as the ground truth to determine the true‐positive fraction, true‐negative fraction, false‐positive fraction, and false‐negative fraction.

Parameters were calculated from the ROC curves to assess the accuracy of the NCM and find the resultant artifact threshold. For each patient in cohort 1, the area under the curve (AUC) was calculated to determine the NCM accuracy, and the Youden index was calculated to extract a threshold. The Youden index J is the maximum vertical distance between the ROC curve and the diagonal “chance line”, which can also be related back to a decision threshold point directly (Eq. (5)).

(5) $$\begin{array}{l} J=\text{max}(sensitivity(dc)+specificity(dc)-1) \end{array}$$

The Youden index represents the optimal cut‐point in ROC curve analysis and is used as another measure of accuracy. Youden's index values vary between 0 and 1, with 1 indicating a relatively large NCM evaluation accuracy.^(28)^ The artifact threshold was derived from the Youden index to provide the optimal artifact threshold corresponding to maximum accuracy in each ROC curve. The point in the curve at which the Youden index was found yielded the corresponding NCM threshold.

The artifact threshold corresponding to the minimum Youden index found in cohort 1 was taken as the final artifact threshold. One outlier index existed, and thus an average of thresholds was deemed inappropriate, and the minimum was taken to ensure that artifacts would not be missed. The determined artifact threshold was applied to each $\left((N_{T-2})\times 10\right)$ matrix of NCM values in cohort 2. All cohort 1 ROC curves and parameters, and cohort 2 sensitivity and specificity values were calculated using consensus group results as the ground truth.

### E. relationship between artifact magnitudes and metric values

To evaluate whether the relative NCM values accurately reflected the degree of artifact severity, or artifact magnitude, the artifact score for each consensus group‐identified artifact among all ten cases was compared to the NCM value corresponding to the couch position and breathing phase of that artifact score. A Pearson correlation coefficient and a p‐value were calculated for each patient.

## III. RESULTS

### A. Metric

**Figure 3:**
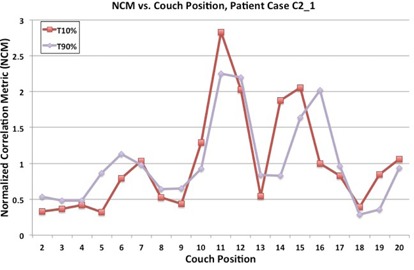
Normalized correlation metric (NCM) vs. couch positions for patient case $C2_{\_}1$. Breathing phases T10% and T90% are displayed for comparison with coronal views of T10% and T90% in Fig. 2.

Evaluated cases are labeled first by the cohort (C1 or C2) followed by a number indicating the order of evaluation within that cohort. An example of NCM values over all couch positions for 2 component phases for the first patient of cohort 2, $C2_{\_}1$, is shown in Fig. 3; coronal image slices from the same 2 component phases of the $C2_{\_}1$ case are also shown in Fig. 2 for comparison to Fig. 3 and for an example of consensus‐chosen scores.

### B. Consensus artifact results

The consensus group scored all 10 phases of a 4D CT scan in under 40 minutes, on average within 30 minutes. The mean percentage of couch positions covering lung anatomy that contained consensus‐chosen artifacts for both cohorts was 68.7%. The first patient scored by the consensus group had the lowest percentage of lung couch positions containing artifacts: 32.4%; all other patients had artifacts in at least 59% of lung couch positions with a maximum percentage of 87.3%.

**Figure 4:**
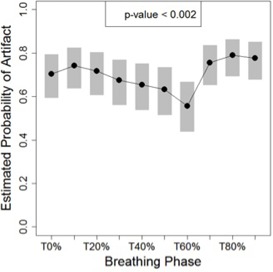
Estimated probability of artifacts as a function of phase and associated 95% confidence intervals (grey bars). The risk of an artifact decreased for exhale phase images. The p‐value derives from the likelihood ratio test of the global null hypothesis of the absence of association with phase.

Artifact incidence was significantly associated with breathing phase using logistic mixed effects analysis (p<0.002 likelihood ratio test). The estimated probability of observing an artifact during the T60% phase was significantly lower than T10% (odds ratio=0.44, p<0.003), T70% (odds ratio=0.41, p<0.0015), T80% (odds ratio=0.33, p<0.0002), and T90% (odds ratio=0.36, p<0.0004) phases after adjusting for multiplicity. Figure 4 demonstrates the model‐estimated probability of an artifact at each phase with 95% confidence intervals.

### C. ROC analysis

**Figure 5:**
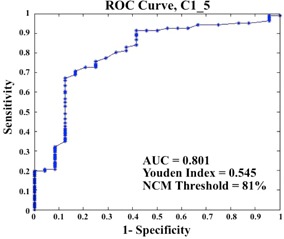
Example ROC curve from case $C1_{\_}5$ of cohort 1, with area under the curve (AUC), Youden index, and corresponding NCM threshold indicated.

Figure 5 demonstrates an example ROC curve generated for case $C1_{\_}5$ with associated parameters, also listed in Table 1, which displays the ROC curve parameters for each cohort 1 patient. The artifact threshold found using this method was 73%. The average (AVG) cohort 2 sensitivity resulting from the applied artifact threshold of 73% was 0.703, and the average specificity of cohort 2 was 0.476, each with a standard deviation (STD) of 0.11.

**Table 1:** Cohort 1 ROC parameters

*Parameter*	$C1_{\_}1$	$C1_{\_}2$	$C1_{\_}3$	$C1_{\_}4$	$C1_{\_}5$
AUC (area under curve)	0.756	0.525	0.769	0.709	0.801
Youden's index	0.446	0.103	0.507	0.461	0.545
NCM threshold	125%	73%	93%	93%	81%

### D. Metric correlation with consensus scores

The mean of cohort 1 Pearson correlation coefficients was 0.80, with all but the first case yielding a coefficient greater than 0.91; the composite correlation coefficient was 0.54. The mean of cohort 2 Pearson correlation coefficients was 0.61, with three of the five cases yielding coefficients greater than 0.99; the composite correlation coefficient was 0.58. The composite p‐values for both cohorts were less than 0.001, indicating a nonzero correlation between the NCM and consensus scores.

## IV. DISCuSSIoN

We found that the average sensitivity and artifact score correlation achieved for the NCM assessment against the consensus group assessment indicate a moderately high performance of the NCM method for ciné 4D CT artifact evaluation. The sensitivity was high, while the specificity was only moderate, suggesting the artifact threshold found using cohort 1 overestimated the true number of artifact locations. This was expected as we chose the artifact threshold corresponding to the minimum Youden index, the cohort 1 outlier, to favor not missing an artifact at the expense of falsely identifying a position as containing an artifact. However, even with this trade‐off, the sensitivity was not as high as expected. This may be due to the inexperience of the consensus group members present with the first few cases. If the consensus group members had participated in a practice session an outlier might have been avoided, and the artifact threshold would have been derived over an average of the sample rather than from the outlier value. Had a larger cohort 1 sample size been evaluated, a decreased outlier detriment would have allowed an average‐derived artifact threshold.

The artifact score correlations between the NCM values and consensus results were good on an individual basis, but a few cases with poor coefficients lowered the composite correlation coefficient. The first case had a poor coefficient (0.169) that would have been improved from a practice session to familiarize the observers with the procedure and appropriate scoring system based on the given instructions. The other two cases containing poor coefficients (0.342 and 0.408) did not contain any consensus magnitude scores higher than 2, which left only two data points for the correlation. An increase in cohort 1 sample size may also have provided more cases with scores over the range 1‐5 to offset these poor correlations.

This study would have benefited from a practice session to train observers before the consensus evaluations and an increased cohort 1 sample size of patient 4D CT cases to offset the effect of an outlier and provide more data points for artifact score correlation. However, even with these weaknesses, the consensus group method of visual assessment allowed an efficient and guided scoring that produced a high quality research dataset for metric validation, a reduced interobserver variation, and provided a more consistent method of identifying artifact locations and magnitudes. The NCM also performed well despite these issues and resulted in a reproducible automated quantitative evaluation within a time unachievable by observer evaluations; the NCM is also simple to implement as it is based on the Pearson correlation coefficient, and thus would integrate well into the clinical workflow.

The results of the logistic mixed effects analysis demonstrate that artifacts are less likely to be present near exhale. This is intuitive as exhale is a more stable breathing state; passive exhale ends at the functional residual capacity, the equilibrium point between the chest wall expansion and lung contraction. In particular, there was a significant reduction in artifact odds associated with T60% when compared to T10%, T70%, T80%, and T90%. These images were phase‐sorted using ten equally divided bins, and did not explicitly define T50% to be maximum exhale. As time from peak inhalation to peak exhalation is typically longer than time from peak exhalation to peak inhalation,^(29)^ T60% may have more truly represented maximum exhale than other phases such as T50%, providing an explanation for why only T60% contained a significant artifact reduction.

Currently, visual assessment is the evaluation standard, but it lacks a clear set of rules, which makes extraction of quantitative results difficult. Some visual assessments display example images with a description of the overall artifact presence, whether on an individual basis or as a comparison between scans,^1^, ^13^, ^30^, ^31^, ^32^, ^33^, ^34^, ^35^, ^36^, ^37^, ^38^ while others state a particular method of an independent expert evaluation.^15^, ^39^, ^40^, ^41^, ^42^ Our study extracted a quantitative artifact evaluation from qualitative observations in a guided and efficient method that reduced interobserver variability. This yielded a high‐quality research dataset for 10 breathing phases of ten patients in approximately 5 hours. This consensus group method of evaluation could also be used for images from various acquisition and processing methods and even for various types of artifacts, as long as instructions on identification and characterization are provided for observer guidance, preferably with a practice session before the first case analysis.

Similar evaluations have been done with images derived from phantoms and software simulations, in which the exact anatomic volume and shape are known.^31^, ^34^, ^35^, ^43^, ^44^, ^45^, ^46^ Persson et al.^14^, ^47^ evaluated artifacts in terms of gross tumor volume deviations in comparison with a reference target volume. Among other quantitative artifact evaluations exist the following: deviations in target centroid position or contours,^37^, ^48^, ^49^ the mean square gray value difference between couch positions,^(1)^ tidal volume variations from reference images,^(36)^ and external surrogate parameters.^(50)^ The quantitative metric evaluated in this study offers an efficient, reliable method to evaluate ciné artifact location and magnitude in the lung.

## V. CONCLUSIONS

We conclude that the correlation metric assessed has the potential to be used as a ciné 4D CT artifact evaluator when an efficient and reliable method is needed for processing many sets of images, though additional cases would yield an even more accurate artifact threshold for identification. We also conclude that the consensus group method has the potential to be used as a research standard for evaluating 4D CT artifacts and as a standard to evaluate alternative quantitative artifact evaluation methods, and recommend a consensus practice session before evaluation.

## ACKNOWLEDGMENTS

We thank the members of the consensus group for their time and participation in the study. Financial support for this research was provided by an NIH Director's New Innovator Award (DP2OD007044). RC was partially supported by NIH through a Loan Repayment Program Award and an NCI Mentored Research Scientist Award (K01CA181292).

## Supporting information

Supplementary Material
