# Supplementary figures and images for: Assessment of a quantitative metric for 4D CT artifact evaluation by observer consensus
Source: J Appl Clin Med Phys. 2014 May 8;15(3):190–201. doi: 10.1120/jacmp.v15i3.4718 (PMC4048877; doi:10.1120/jacmp.v15i3.4718)

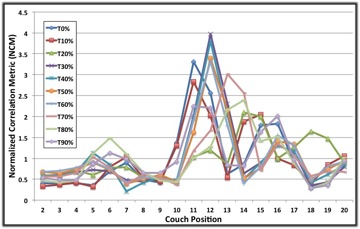

Supplement: Supplementary file 1 — Supplementary Material [file ACM2-15-190-s001.jpg]
